# An assessment of Veterans attitudes and willingness to receiving the COVID-19 vaccine: a mixed methods study

**DOI:** 10.1186/s12879-022-07269-7

**Published:** 2022-03-29

**Authors:** Jessica Gardner, Gabriel Brown, Jadisha Vargas-Correa, Frances Weaver, Israel Rubinstein, Howard S. Gordon

**Affiliations:** 1 Jesse Brown Veterans Affairs Medical Center, 820 S. Damen Ave (151), Chicago, IL 60612 USA; 2 VA Center of Innovation for Complex Chronic Healthcare, Chicago, IL USA; 3 Parkinson School of Health Sciences and Public Health, Loyola University Chicago, Chicago, USA; 4 Division of Pulmonary, Critical Care, Sleep, and Allergy Medicine, Department of Medicine, University of Illinois at Chicago, Chicago, IL USA; 5 Division of Academic Internal Medicine, Department of Medicine, University of Illinois at Chicago, Chicago, IL USA; 6 Institute for Health Research and Policy, University of Illinois at Chicago, Chicago, IL USA

**Keywords:** Vaccine Hesitancy, Survey Research, Qualitative Research, Veterans, Trust in healthcare, Acceptance of healthcare

## Abstract

**Background:**

While several safe and effective COVID-19 vaccines have been available since December 2020, many eligible individuals choose to remain unvaccinated. This vaccine hesitancy is an important factor affecting our ability to combat the COVID-19 pandemic.

**Methods:**

The objective of the study was to examine the attitudes and willingness among US Veterans toward receiving COVID-19 vaccination. The study used a quantitative qualitative mixed methods design with a telephone survey and then in-depth interviews in a subset of those surveyed. Participants were unvaccinated Veterans (N = 184) selected randomly from a registry of patients who had received VA healthcare during the pandemic and had a diagnostic test for COVID-19. The primary outcome was willingness to accept COVID-19 vaccination. Survey data collection and in-depth interviews were conducted by telephone. Analyses of the survey data compared the primary outcome with demographics, clinical data, and survey responses using bivariate and multiple regression analyses. A subset (N = 10) of those surveyed, participated in an in-depth interview. Interview transcripts were analyzed to derive themes using qualitative content analysis.

**Results:**

Almost 40% of participants disagreed they would receive a COVID-19 vaccine. Participants who were younger, female, and had fewer comorbid conditions were more likely (P < 0.05) to disagree with COVID-19 vaccination. In multiple regression analysis, willingness to accept vaccination was associated with reliance on a doctor or family member’s recommendation and with a belief that vaccines are effective. In-depth interviews revealed several barriers to COVID-19 vaccination, including lack of trust in the government and vaccine manufacturers, concerns about the speed of vaccine development, fear of side effects, and fear the vaccine was a tool of racism.

**Conclusions:**

This study illustrates the complexity of patients’ deliberation about COVID-19 vaccination and may help physicians and other health care providers understand patients’ perspectives about COVID-19 vaccination. The results highlight the importance of patients’ trust in physicians, healthcare organizations, pharmaceutical manufacturers and the government when making health decisions.

**Supplementary Information:**

The online version contains supplementary material available at 10.1186/s12879-022-07269-7.

## Background

Despite the widespread availability of three safe and effective COVID-19 vaccines in the US many eligible individuals choose to remain unvaccinated for various reasons [1–4]. This vaccine hesitancy or deliberation is an important factor affecting our ability to combat the ongoing COVID-19 pandemic.

Although there is agreement that an increase in COVID-19 vaccine uptake is needed to achieve herd immunity [5], efforts to increase vaccination rates face several barriers. As described in several populations, vaccine hesitancy—which is driven in part by rumors, misinformation, and distrust—was associated with female sex, less education, unemployment, lower income, black race, having children at home, and perceived threat of contracting COVID-19 in the next year [1–4]..

While there are several studies reporting the general population’s willingness to accept a COVID-19 vaccine, few studies have examined these attitudes among U.S. Veterans. Capturing the sentiment toward a COVID-19 vaccine among Veterans is important because over 50% of the US Veteran population is aged 65 years or older. Further, Veterans have a higher prevalence of multiple chronic health conditions such as hypertension, diabetes mellitus, COPD, and cancer compared to the general U.S. population placing them at higher risk of severe illness and mortality from COVID-19 [6, 7]. Such information can be useful to determine what COVID-19 vaccine strategies might be deployed to improve COVID-19 vaccine acceptance. We used a combination of a telephone survey and in-depth interviews to assess Veterans’ perceptions and attitudes associated with willingness to accept a COVID-19 vaccine. Moreover, eligible Veterans for this study were predominantly African Americans who reside in inner city Chicago neighborhoods that were hit hard during the COVID-19 pandemic.

## Methods

### Study population

Participants were recruited from the Jesse Brown Veterans Affairs Medical Center (JBVAMC) Registry for Research on Risk Factors and Outcomes of Veterans Evaluated for COVID-19 (the Registry). At study onset, the Registry included all JBVAMC patients who had a positive, presumptive positive, or negative diagnostic test for COVID-19 between March and December 2020. Data for recruitment were obtained from the VA Corporate Data Warehouse curated by the VA Shared Data Resource and from the VA Computerized Patient Record System (CPRS). The study was approved by the JBVAMC Institutional Review Board and Research and Development Committee.

### Survey development

We used the Health Belief Model and Ecological Model to guide the development of a survey about willingness to accept vaccination for COVID-19 [8, 9]. The Health Belief Model (HBM) has six main constructs: perceived susceptibility, perceived severity, perceived benefits, perceived barriers, self-efficacy to engage in behavior and cues to action [8]. Perceived susceptibility refers to an individual’s beliefs about their vulnerability to a specific disease or condition. Perceived severity is their belief about how their health will be impacted. Perceived benefit(s) refers to positive outcomes associated with taking a certain measure, while perceived barriers encompass factors that prevent a person in taking part in that measure. Self-efficacy refers to a person’s ability to engage in a behavior and cues to action refer to information, people and events that trigger a response toward a behavior. While the HBM takes an intrapersonal approach to understanding and predicting behavior, the Ecological Model’s attention to individual and environmental determinants of behavior provides additional dimensions (intrapersonal, interpersonal, organizational, community and public policy) that may be considered when examining individuals’ decisions to get vaccinated [9].

Using constructs from the HBM and Ecological models, we designed a survey to collect information on several potential predictors of vaccine perception (e.g. age, race, and education), health status [10], awareness and knowledge of COVID-19, attitudes and beliefs regarding vaccination, and trust in health care providers and the Veteran Healthcare System [11, 12]. Previous literature assessing vaccine perceptions for influenza and COVID-19 were reviewed to identify additional areas for the survey [1–3, 8, 13, 14]. The survey was also designed to collect information on Veterans’ preferred method of receiving information about a COVID-19 vaccine to aid their decision-making process. We used reporting guidelines from the Standards for Reporting Qualitative Research (SRQR) and the Strengthening the Reporting of Observational Studies in Epidemiology (STROBE) as aids to presenting this work (Additional file 3: Table S1 and Additional file 4: Table S2).

### Recruitment

Eligible Veterans were invited to participate in the telephone survey after review of CPRS indicated they lived independently, had provided an address and phone number, and had not already received a COVID-19 vaccine. Target enrollment was no more than 200 Veterans for the survey and 10 Veterans for the in-depth interviews based on available funding. Eligible participants were chosen randomly from the Registry and were mailed an opt-out, recruitment letter and information sheet informing them of the study and asking them to call the research staff if they did not wish to participate. Those who did not opt out were called by the research staff 7–10 days after mailing the letter. Veterans who consented to participate in the survey were also asked if they would agree to participate in an in-depth interview about COVID-19 vaccination. Veterans who disagreed or strongly disagreed with the question, “Once a vaccine for COVID-19 becomes available to me at the VA, I will get it” were invited to participate in the in-depth interview. Interactions with participants were conducted by phone or with teleconferencing software and all participants provided verbal consent.

### Data and measures

Participants’ age, sex, race/ethnicity were determined from computerized patient medical records. Participants answered questions about their education level, primary mode of transportation, living situation, mental and physical health status [10], screeners for depression and anxiety [15, 16], trust in provider and VA healthcare [11, 12] and trust in the US government’s management of the pandemic, knowledge about COVID-19, and sentiment toward vaccines. The primary outcome was the response to a question about willingness to receive a COVID-19 vaccine when available at the VA. The full version of the survey is available in Additional file 1: Figure S1.

Semi-structured telephone interviews were conducted using an interview guide to elicit the Veterans’ experiences with the COVID-19 pandemic and their sentiments towards vaccines in general and towards receiving a COVID-19 vaccine. Interviews were audio-recorded and transcribed verbatim by research staff. The full interview guide can be found in Additional file 2: Figure S2.

### Quantitative analysis

We assessed willingness to accept a COVID-19 vaccine by categorizing Veterans who strongly agreed or agreed compared with those who disagreed or strongly disagreed with the primary outcome question “When a vaccine is available at the VA, I will get it.” We estimated bivariate associations between vaccine willingness and participant characteristics, knowledge of COVID-19 pandemic, perceived risk of and perceived severity of COVID-19 infection, sentiment toward vaccines, trust in provider, in the VA, and the government with Chi-square tests for categorical variables and non-parametric tests or T-tests for continuous variables. We used logistic regression to examine independent predictors of willingness to accept a COVID-19 vaccine using backward elimination of demographic and clinical characteristics, and responses to survey items and scales that were statistically significant P < 0.05 and keeping variables with P < 0.15 in the regression model. Data were collected and managed using REDCap and SAS v9.4 was used for quantitative analyses.

### Qualitative analysis

We conducted a qualitative content analysis to derive themes and codes from interview transcripts [17]. The qualitative analysis began after the first interview and continued through the last interview. Two coders used open coding to identify initial thematic categories and develop the initial codebook. All transcripts were coded independently by up to five coders. Initial codes were edited, and additional categories were added to the codebook. Coders met to refine the codes and to resolve any disagreement in coding and to group codes into thematic categories. Strength of the interpretations was achieved with triangulation of multidisciplinary (medicine, psychology, public health) in conjunction with multicultural perspectives among the coders. Quotes were labeled with an anonymized numerical code for each participant.

## Results

### Telephone vaccine survey

**Fig. 1:**
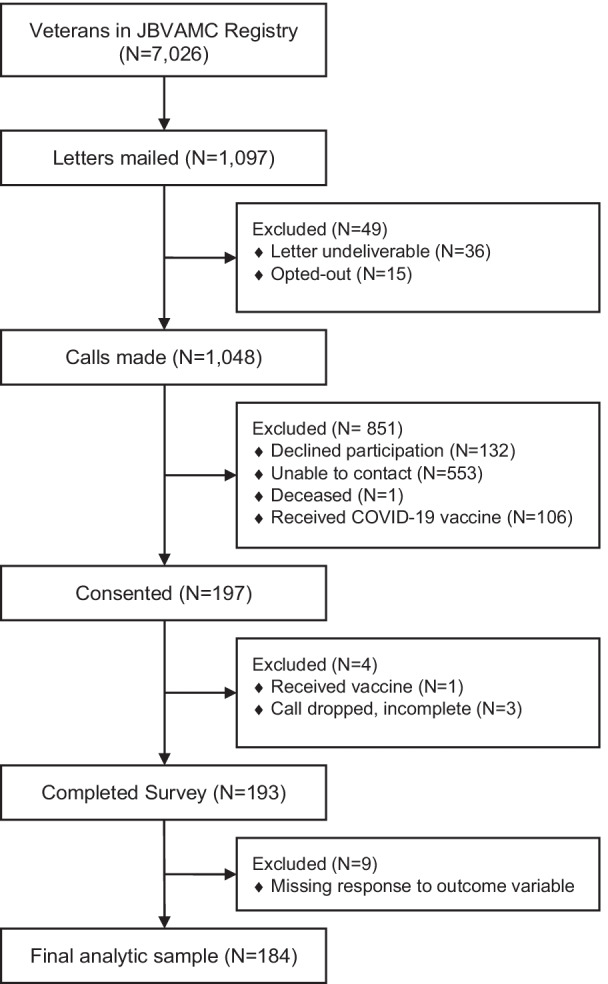
Vaccine Survey Enrollment December 2020 through June 2021

Of those invited to participate, 197 Veterans consented to participate in the telephone survey (see Fig. 1 for recruitment flow diagram). After excluding 13 respondents with missing data for the dependent variable (intention to vaccinate), a total of 184 observations were considered for analysis. Overall, 60% (N = 111) of Veterans agreed or strongly agreed that they would receive the COVID-19 vaccine once available to them at the VA.

Veterans who disagreed with the statement that they would receive an available COVID-19 vaccine were younger (51.6 vs 58.7 years; P = 0.0003), more likely to be female (28.8 vs 9%; P = 0.0005), less likely to live alone (P = 0.03); less likely to use public transportation (P = 0.01) and had fewer comorbid conditions (1.9 vs 3.0; P = 0.02 and 8.3 vs. 12.7; Charlson and Elixhauser scores respectively; Table 1). There was no significant difference in responses about generalized anxiety (P = 0.09) nor in rating of physical functioning (P = 0.96). However, Veterans who disagreed they would accept an available COVID-19 vaccine had higher scores on the depression screener (2.5 vs 2.0; P = 0.02) and lower self-ratings of mental health status (38.5 vs 45.1; P = 0.002) compared with participants who agreed to get vaccinated.

**Table 1:** Demographic and clinical characteristics of study participants

Characteristic	Will get a vaccine once available		P-value
	Agree [N = 111]	Disagree [N = 73]	
	*% (N)*	*% (N)*	
Male Sex	91.0 (101)	71.2 (52)	**0.0005**
African American/Black [N = 182]	70.6 (77)	63.0 (46)	0.28
Hispanic/Latinx [N = 183]	9.0 (10)	8.3 (6)	0.87
Married	35.1 (39)	31.5 (23)	0.61
College degree	28.8 (32)	26.0 (19)	0.68
Single family residence	47.8 (53)	60.3 (44)	0.10
Live alone [N = 183]	35.5 (39)	20.6 (15)	**0.03**
Primarily use public transportation	24.3 (27)	9.6 (7)	**0.01**
	*Mean (SD)*	*Mean (SD)*	
Age (Mean years, SD)	58.7 (12.4)	51.6 (13.1)	**0.0003**
PHQ-2* Score† (Mean Score)	1.8 (1.9)	2.5 (2.0)	**0.02**
GAD-2* Score† (Mean Score) [N = 180]	2.0 (2.0)	2.5 (2.3)	0.09
VR-12* Physical Component Score‡ (Mean, SD)	35.5 (11.5)	35.4 (13.7)	0.96
VR-12* Mental Component Score‡ (Mean, SD)	45.1 (13.0)	38.5 (15.0)	**0.002**
Comorbidity Index (Charlson)	3.0 (3.02)	1.9 (2.5)	**0.02**
Comorbidity Index (Elixhauser)	12.7 (14.8)	8.3 (5.5)	**0.04**

^*^ PHQ denotes Patient Health Questionnaire; GAD denotes Generalized Anxiety Disorder questionnaire; and VR-12 denotes the short form of the Veterans Rand -12 questionnaire

^†^Score range is 0 to 6 points

^‡^Score normalized from 0 to 100 points

Bolded emphasis indicates statistical significance at P < 0.05

Participants’ ratings of their own knowledge about COVID-19 illness or transmissibility did not differ (P > 0.05; Table 2) among those agreeing or disagreeing that they would get vaccinated. Knowledge about symptoms and signs of COVID-19 infection including fever, cough, shortness of breath, headache, myalgia, fatigue, and loss of taste or smell were similar in both groups (P > 0.05, not shown). Those who did not agree to get an available COVID-19 vaccine had lower (P = 0.04) perceived risk of being infected with COVID-19. Yet, most participants who agreed or disagreed to get a COVID-19 vaccine (75.5% vs 69.6%; P > 0.05) perceived that severity of disease would be high if infected. Participants who rated vaccines in general as safe and effective were significantly more likely to agree to get an available COVID-19 vaccine (P < 0.001). Additionally, those who disagreed with receiving a COVID-19 vaccine indicated they did not get the annual influenza vaccine (P = 0.0001). Participants indicated that recommendations from a doctor, religious leader, or family member would be influential in agreeing to be vaccinated with an available COVID-19 vaccine (P < 0.0001; Table 2).

**Table 2:** Knowledge and Beliefs about COVID-19 and COVID-19 vaccines

Knowledge or Belief	Will get a vaccine once available		P-value
	*Agree* *N* = *111*	*Disagree* *N* = *73*	
	*% (N)*	*% (N)*	
Perceived knowledge about COVID-19 pandemic (Moderate/A lot) [N = 183]	78.2 (86)	80.8 (59)	0.67
**Agree that:**			
Persons ≥ 65 are more likely to get more severe illness from COVID-19 [N = 181]	95.4 (103)	91.8 (67)	0.32
COVID-19 can be spread from person-to-person	99.1 (108)	95.9 (70)	0.15
People with COVID-19 always show symptoms	19.6 (21)	9.70 (7)	0.07
Most people who get COVID-19 only show mild symptoms [N = 174]	42.3 (44)	34.3 (24)	0.29
After a person has recovered from COVID-19 he/she cannot get it again [N = 179]	7.3 (8)	5.7 (4)	0.67
Low perceived risk of personally getting COVID-19 [N = 181]	36.4 (40)	52.1 (37)	**0.04**
High perceived severity if infected with COVID-19 [N = 171]	75.5 (77)	69.6 (48)	0.39
**I believe that:**			
Vaccines are safe for the general population [N = 177]	86.2 (94)	52.9 (36)	** < 0.0001**
There is enough information about vaccine safety [N = 181]	68.7(76)	30.6 (22)	** < 0.0001**
Vaccines decrease the chance of infections [N = 178]	83.3 (90)	60.0 (42)	**0.0005**
Vaccines decrease the severity of disease if infected [N = 174]	87.6 (92)	63.8 (44)	**0.0002**
Vaccines make me less worried about becoming infected with diseases N = 177]	79.4 (85)	34.3 (24)	** < 0.0001**
Never receive the annual flu shot	15.3 (17)	45.2 (33)	** < 0.0001**
Soreness (in the arm) would make me less likely to get the vaccine [N = 183]	14.4 (16)	26.4 (19)	**0.04**
**Agree with: I will get the COVID-19 vaccine if**			
A doctor recommends it for me [N = 183]	91.0 (101)	12.5 (9)	** < 0.0001**
A religious leader recommends it for me [N = 174]	61.4 (62)	4.1 (3)	** < 0.0001**
A family member recommends it for me [N = 178]	78.9 (86)	4.4 (3)	** < 0.0001**
Would recommend friends/family get the COVID-19 vaccine [N = 181]	83.6 (92)	14.1 (10)	** < 0.0001**

Bolded emphasis indicates statistical significance at P < 0.05

Veterans who disagreed with getting an available COVID-19 vaccine indicated statistically significantly lower trust in US government’s management of the pandemic; lower trust in physicians/health care providers, and lower trust in the VA healthcare system, compared with those who agreed to be vaccinated with an available COVID-19 vaccine (P < 0.001; Table 3).

**Table 3:** Participant ratings of trust

	Will get a vaccine once available		P-value
	Agree Mean (SD)	Disagree Mean (SD)	
General Trust in Physician	78.6 (20.8)	66.6 (22.4)	**0.0003**
General Trust in VA Healthcare (N = 183)	79.0 (19.9)	64.4 (23.4)	**0.0001**
Trust government management of pandemic	42.0 (20.2)	20.9 (24.2)	**0.0001**

Bolded emphasis indicates statistical significance at P < 0.05

We examined the independent relationship of agreement to be vaccinated with an available COVID-19 vaccine outcome with demographic, clinical, and questionnaire variables that were significant in the bivariate analysis using backward stepwise logistic regression and included a race variable in the final model. Agreement to be vaccinated with an available COVID-19 vaccine did not differ by age in years (OR 1.05; 95% CI 1.00–1.10) or black vs other races (OR 1.05; 95% CI 0.25–4.37) but was associated with significantly higher odds for vaccination recommended by a doctor (OR 16.9; 95% CI 4.9–58.1), by a family member (OR 26.0; 95% CI 5.4–125.8), and for those indicating agreement with the statement that vaccines make me less worried about becoming infected with diseases (OR 5.1; 95% CI (1.4–18.8).

### In-depth interviews of 10 participants disagreeing to get COVID-19 vaccination

Participants who disagreed they would get a vaccine for COVID-19 once it becomes available were invited to participate in a telephone interview. Of the 10 interviewed, 9 identified as Black/African American, one as Hispanic/Latinx, 6 were male, and the mean age was 52.8 years (range from 27 to 73 years). We identified several codes associated with COVID-19 and vaccination from the interviews. These results are limited to codes that identify sentiments expressed by at least 4 of the interviewees. Codes were categorized into six themes that could contain more than one code. Quotes representing the themes—effects of COVID-19 pandemic; experiences with vaccines; and barriers to COVID-19 vaccination—are presented below. Additional quotes in Table 4 represent the theme barriers to COVID-19 vaccination and the themes decision-making for receiving or not receiving the COVID-19 vaccine; use of COVID-19 vaccination as a tool of racism; and cues to action.

**Table 4:** Themes, codes, and illustrative quotations about COVID-19 and vaccination

**Theme I: Barriers to COVID-19 vaccination**	
**Speed of development**	*“But the only reason why I’m skeptical is because of how quickly they came out with the vaccine.” [#6]*
**Risk of side effects**	*“…but if I’m not feeling it then why put myself through the getting the [COVID-19] shot, and having a day where you don’t feel so great… why put my body through the stress?” [#3]*
	*“If 6 months down the line they [other people] are experiencing headaches, or there’s anything degenerative in their overall health that wasn’t there prior to taking the COVID vaccine. Are they experiencing things that they think may be attributed to receipt of the vaccine?” [#5]*
**Theme II: Decision making for receiving or not receiving the COVID-19 vaccine**	
**Deliberation**	*“They might come out with a better vaccine, but you can’t have both of them… The better one then, I would have to wait some more I guess to see if it works.” [#1]*
	*“Again, here I am skeptical and hesitant. And I don’t know which one to take, number one. I don’t know which one would be better for me. The Pfizer might be good for you. The Johnson might be good for [name]… There’s a variety; all three of them gives you a choice of which one to take. But how do I know which one is best for me? … I just don’t know what to do…which way to go. I’m undecided, and I don’t know what particular criteria to use to make a choice.” [#4]*
**Active postponement**	*“…the vaccine is tricky, that’s why I haven’t taken a shot yet…I want to wait and see what it’s going to do to other people in 3 months—6 months…” [#1]*
	*“They want me to take it…I told them I will when I feel comfortable with it, but—not too soon and I don’t need to be in the first wave of people taking it.” [#3]*
	*“…until they make it mandatory, and I have the choice, I am going to wait. Nothing personal to the people who have created these vaccines. Or to the doctors that recommend it, or to you, your staff, anyone. I am just not sure.” [#4]*
	*“Well, it could be forever—I may decide that I don’t want it at all. But at this point I am open because at the early stages of the pandemic announcement my daughter was sick, and she was sick for 28–29 days and it really did her bad. So, I know that it is real, but I don’t know if I’m ready to subject myself to the vaccine.” [#5]*
**Theme III: Use of COVID-19 vaccination as a tool of racism**	
	*“… I’m speaking from a black perspective—most of my friends… and a lot of black people are afraid that there might be two different vaccines out there. One that is geared toward White Americans and one that is geared toward Black Americans. A lot of people are afraid that thing administered to African Americans might not be the same thing that they are administering to White Americans and it could have an adverse effect on African Americans.” [#5]*
**Theme VI: Cues to Action**	
**Repercussions of not getting vaccinated**	*“You can do more if you got a shot than somebody can do if they don’t get the shot. It’s almost like let’s vilify the person that’s not gonna get it. And the people that do get it, they’re good.” [#10]*
	*“… the state or the government would have to regulate it to say that they had to have that [the COVID-19 vaccine]. They’re not going to say, “Hey, you, you have to go get your shot”. They’re just going to say, “You need this shot if you’re going to continue work.” [#2]*
	*“I’m open to it. I’m open to see. But it has to be something that’s going to be convincing to me. That it’s necessary. And that if I don’t take it, it’s going to cause repercussions in some kind of way, that’s going to require me to have to take it. Otherwise, I don’t be able to do z-y–z, or I won’t be able to see my grandchildren; I won’t be able to go back to church; or I won’t be able to go to the store anymore.” [#4]*
**Peer or social network influence**	*“Cause if I voice my personal opinion to a so-called friend to how I feel about it [the COVID-19 vaccine] then our friendship is ruined.” [#9]*
	*Well, every now and then I’ll go online… but… I’m more concerned about people that I actually know; how their faring after taking it…those are the people I’m going to really rely on—folks that I see and talk to on a regular basis.” [#5]*

### In-depth interviews: Selected themes and example quotations

Participants highlighted negative effects of the COVID-19 pandemic on their mental health. One participant who worked in a funeral home commented:

*“That’s one of the things that’s really has affected me—the deaths that I’ve seen… That’s a strain on my mind.” [#4]*

Another participant who had frequent job-related interactions with others indicated concern about getting COVID-19:

*“…I’m so paranoid about it. It’s like every two weeks I go get tested, just for reassurance.” [#7]*

All the participants were US Veterans, and most described experiences or sentiments about vaccination that were related to their military experience. There was general acceptance of receiving vaccines while on active military duty because of a trust or faith the armed services had a mutual interest in service members’ health:

*“…you don’t get to choose what vaccines you get in the military… you wouldn’t think that the military—the people that are paying you to go and do something to put your life on the line to protect and serve the country—are gonna screw you up medically so you can’t do that. It’s like you have faith in them, because they got faith in you to do a job… you’re givin’ up those rights an organization that’s gonna have your best interests because they need, they need you.” [#10]*

Experiences with vaccination after military service differed across participants:

*“I haven’t taken any shots since I left the military.” [#7]*

*“…if it weren’t for the doctor’s recommendation, I wouldn’t be considering it [the flu shot] at all… that’s the only reason I got one this year.” [#8]*

Participants described several lines of reasoning that fit a theme of barriers to vaccination. A common sentiment was a distrust in the COVID-19 vaccine and that pharmaceutical manufacturers were experimenting on them:

*“I’m not going to be anybody’s test dummy… everybody is a test dummy—they don’t know what effects are going to happen and I need to see some results after a year of everybody getting it [the COVID-19 vaccine]; if those people are still even alive” [#9]*

Participants described an accumulated distrust in government treatment of African Americans with mention of the Tuskegee study, and that lack of confidence was reinforced by frequent changes in recommendations (e.g., about mask wearing) as expressed in this quote:

*“And then I think the biggest thing, the biggest thing that makes me not trust the vaccine… for them to be them to be just up-and-down, left and right, with the guidances that they put out, it makes no sense. It almost feel-feels like they’re lying.” [#10]*

See Table 4 for additional quotes representing barriers to COVID-19 vaccination.

## Discussion

In this mixed methods study we conducted telephone surveys and in-depth interviews with Veterans from a single tertiary care, academically affiliated VA Medical Center. Of the 184 participants in the telephone survey almost 40% disagreed they would receive a vaccine for COVID-19 once available to them at the VA. Disagreement was associated with being younger, female, with fewer number of comorbid medical diagnoses, and with higher mean depression scores and lower mental health scores. Veterans disagreeing to get a vaccine were less likely: to agree vaccines are safe and effective; to get an annual influenza vaccination; to follow recommendations from others to get vaccinated; and had less trust in physicians, VA healthcare, and the government. In-depth interviews of participants who disagreed with getting vaccinated added a detailed perspective on Veterans reasons for not getting COVID-19 vaccination. Our results highlight the complexity of patients’ deliberation about COVID-19 vaccination and may help physicians and other health care providers who are recommending COVID-19 vaccination to understand patients’ perspectives. Our results that recommendations from a family member or a personal physician and a belief that vaccines are protective against disease were independent predictors of COVID-19 vaccination and that demographic factors are not significant independent predictors might be useful to policy makers and health care workers considering how to improve vaccination rates.

These data are novel in the collection of several clinical factors and our linkage of survey data to clinical data from the electronic medical record. We found that self-reported scores of physical functioning and generalized anxiety were no different among Veterans who agreed or disagreed to get a COVID-19 vaccine, but those who disagreed they would get vaccinated had statistically significantly lower scores on mental function, higher scores on a depression screener, and had fewer comorbid conditions.

Many previous studies examining vaccine hesitancy were conducted before COVID-19 vaccines became available in the US [1–4, 8, 18–25]. Many of these studies examined constructs from the Health Belief Model (e.g., perceived susceptibility, benefits, and barriers) and the Ecological Model (e.g., intrapersonal, interpersonal, institutional, and community-level factors) that affect intentions to get a COVID-19 vaccination [8, 18, 25, 26]. Reported results from survey/questionnaires indicated that individuals who would not agree to vaccination were younger [2–4, 19, 21, 22], more likely to be female [3, 4, 19, 22, 23], more likely to mistrust the government [19, 22], were less likely to think vaccines are safe and effective [19, 22], and less likely to get a vaccine when a doctor recommends it [22]. Several studies of willingness to get vaccinated for COVID-19 used self-report survey data to collect and report about participants health conditions [20, 24], which differs from our use of data from electronic medical records to assess comorbidity.

Our qualitative results offer a novel perspective on Veterans’ decision-making process when considering COVID-19 vaccination. In contrast to their willingness to accept vaccinations during their military service, a legacy of distrust of government and healthcare led these Veterans to remain undecided about whether or when they would accept COVID-19 vaccination. Instead, most indicated they were not refusing vaccination, but were waiting to decide about vaccination, sometimes indicating they were watching to see what happened to others who were vaccinated. Yet, these comments revealed an internal conflict between acceptance and refusal evidenced by the indeterminate time frame for when these Veterans would be ready to get a vaccine.

Concerns expressed about the role of racism in efforts by the US government and others to encourage immunization against the COVID-19 vaccine were also novel. Though we found no significant difference by race or ethnicity in agreement to accept a vaccine in those completing the questionnaire, the Veterans who were interviewed were concerned that Black Americans were treated differently regarding vaccination. Concerns about targeting of vaccination in predominantly black communities included a belief that there were two vaccines. Specifically, beliefs that health care facilities in black communities would receive a different vaccine than those in white communities, and that the vaccine targeted to Black Americans would have adverse effects on their health. Thus, participants were concerned the COVID-19 vaccine was a tool of racism.

Our qualitative findings regarding differences underlying willingness to accept vaccines in the military and hesitancy as civilians and regarding concerns the vaccine is a tool of racism are both, to our knowledge, unique findings. In addition, our findings are consistent with qualitative studies conducted prior to the availability of vaccines. Several qualitative studies involving black communities found participants expressed concerns about the speed of vaccine development, vaccine safety, and mistrusted government and healthcare initiatives [27–29]. A qualitative study that interviewed Veterans and VA staff in three VA medical centers after COVID-19 vaccines became available found several similar concerns to those we report including mistrust of government, though half of the interviewees had received at least one COVID-19 vaccine [30]..

Our study had several strengths, including the supplementation of survey data with data from the medical record, mixed quantitative–qualitative methods, in-depth interviews of a subset of respondents and a patient population that is largely African American. Members of this group are often underrepresented in research and have been disproportionately affected by the COVID-19 pandemic [31]. Our study results also should be considered in the context of several limitations. First, our study population was small and limited to patients who received care at a single VA medical center and may not generalize to other populations. Second, our qualitative interviews were conducted with ten Veterans, and we may not have achieved saturation of themes—however our interviews had features consistent with sufficient information power [32]. That is, our interviews included participants who were homogeneous (unvaccinated Veterans), our interviews included rich responses, our aims were narrowly focused on COVID-19 and vaccination, and the interviews were guided by established theories of patient acceptance of health care.

## Conclusions

Our findings highlight certain demographic and health characteristics that are associated with willingness to accept a COVID-19 vaccine. Race was not associated with willingness to get a COVID-19 vaccination, while age, gender, mental health, and number of comorbid conditions were associated with willingness to accept a vaccine. However, these characteristics became statistically insignificant when compared to the effect of a belief in the effectiveness of vaccines and the effect of recommendations from trusted physicians or providers and family members. These results highlight the importance of trusted relationships with a health care provider when considering health care interventions such as vaccination. Patient relationships with a physician or provider and patients’ trust in the physician may not be independent of the practice setting. Health decisions also depend on trust in healthcare organizations including hospitals, pharmaceutical manufacturers, and the government. Organizational and governmental efforts that effectively communicate and present information about vaccines should improve knowledge dissemination about vaccines and institutional policies that support patients’ development of trusting relationships with physicians and providers, such as policies to maintain continuity of care with a health care provider, allow patients to develop, build, and sustain trust in healthcare.

## Supplementary Information

**Additional file 1:** Telephone Survey.

**Additional file 2:** In-depth interview guiding questions.

**Additional file 3:** Reporting guidelines for Standard for Reporting Qualitative Research (SRQR).

**Additional file 4:** Reporting guidelines for Strengthening the Reporting of Observational Studies in Epidemiology (STROBE).

## Data Availability

Upon request, the corresponding author will consider requests for the final dataset. These requests for access will be reviewed by the Jesse Brown VA Research and Development Committee and Associate Chief of Staff for Research and addressed within a reasonable timeframe. The dataset will include deidentified data relevant to the specific request.

## Abbreviations

CPRS: Computerized Patient Record System
GAD: Generalized Anxiety Disorder questionnaire
HBM: Health Belief Model
JBVAMC: Jesse Brown Veterans Affairs Medical Center
PHQ: Patient Health Questionnaire
SD: Standard Deviation
US: United States
VA: United States Department of Veterans Affairs
VR-12: Veterans Rand -12 questionnaire
