# Supplementary material for: An assessment of Veterans attitudes and willingness to receiving the COVID-19 vaccine: a mixed methods study
Source: BMC Infect Dis. 2022 Mar 29;22:308. doi: 10.1186/s12879-022-07269-7 (PMC8961481; doi:10.1186/s12879-022-07269-7)
Supplement: Supplementary file 1 — Additional file 1: Telephone Survey. [file 12879_2022_7269_MOESM1_ESM.pdf]

Health Status				
**Item #**	**Construct**	**Item**	**Response Scale**	**Interviewer Notes**
**N/A**	Introduction	The purpose of this survey is to assess Veteran's attitudes and perception of the new COVID-19 vaccine. Your participation will inform how the VA and its providers will share information related to the new COVID-19 vaccine. You may decline to answer any questions that make you feel uncomfortable. We appreciate you taking time out of your day to help us. This survey is estimated to take 30-45 minutes, please let me know when you are ready to begin.	**N/A**	**N/A**
**N/A**	Transition	The first set of questions will pertain your overall health.		
**H1**	RAND-12 (VR-12)	In general, would you say your health is:	1= Excellent<br>2= Very good<br>3= Good<br>4= Fair<br>5= Poor	
**N/A**	RAND-12	The following questions are about activities you might do during a typical day. Does your health now limit you in these activities? If so, how much?	N/A (transition)	
**H2A**	RAND-12	Moderate activities, such as moving a table, pushing a vacuum cleaner, bowling or playing golf?	1= Yes, limited a lot<br>2= Yes, limited a little<br>3= No, not limited at all	
**H2B**	RAND-12	Climbing several flights of stairs?	1= Yes, limited a lot<br>2= Yes, limited a little<br>3= No, not limited at all	
**N/A**		During the past 4 weeks, have you had any of the following problems with	N/A (transition)	

Jesse Brown VAMC IRB
Approval Date: December 23, 2020

		your work or other regular activities as a result of your **physical health**?	
**H3A**	RAND-12	Accomplished less than you would like.	1= No, none of the time<br>2= Yes, a little of the time<br>3= Yes, some of the time<br>4= Yes, most of the time<br>5= Yes, all of the time
**H3B**	RAND-12	Were limited in the kind of work or other activities	1= No, none of the time<br>2= Yes, a little of the time<br>3= Yes, some of the time<br>4= Yes, most of the time<br>5= Yes, all of the time
**N/A**	(transition)	During the past 4 weeks, have you had any of the following problems with your work or other regular activities as a result of any **emotional problems**?	N/A(transition)
**H4A**	RAND-12	Accomplished less than you would like.	1= No, none of the time<br>2= Yes, a little of the time<br>3= Yes, some of the time<br>4= Yes, most of the time<br>5= Yes, all of the time
**H4B**	RAND-12	Didn't do work or other activities as carefully as usual.	1= No, none of the time<br>2= Yes, a little of the time<br>3= Yes, some of the time<br>4= Yes, most of the time<br>5= Yes, all of the time
**H5**	RAND-12	During the past 4 weeks, how much did pain interfere with your normal work (including both work outside the home and housework?)	1= Not at all<br>2= A little bit<br>3= Moderately<br>4= Quite a bit<br>5= Extremely
**N/A**	Transition	These questions are about how you feel and how things have been with you during the past 4 weeks. For each question, please give one answer that comes closest to the way you have been feeling.	N/A (transition)
**H6A**	RAND-12	How much of the time during the past 4 weeks:<br><br>Have you felt calm and	1 = All of the time<br>2= Most of the time<br>3= A good bit of the time<br>4= Some of the time

Jesse Brown VAMC IRB
Approval Date: December 23, 2020

		peaceful?	5= A little of the time<br>6= None of the time
**H6B**	RAND-12	How much of the time during the past 4 weeks:<br><br>Do you have a lot of energy?	1 = All of the time<br>2= Most of the time<br>3= A good bit of the time<br>4= Some of the time<br>5= A little of the time<br>6= None of the time
**H6C**	RAND-12	How much of the time during the past 4 weeks:<br><br>Have you felt downhearted and blue?	1 = All of the time<br>2= Most of the time<br>3= A good bit of the time<br>4= Some of the time<br>5= A little of the time<br>6= None of the time
**H7**	RAND-12	During the past 4 weeks, how much of the time has your physical health or emotional problems interfered with your social activities (like visiting friends, relatives, etc.)	1= All of the time<br>2= Most of the time<br>3= Some of the time<br>4= A little of the time<br>5= None of the time
**N/A**	Transition	Now, we'd like to ask you some questions about how your health may have changed.	N/A; transition
**H8**	RAND-12	Compared to one year ago, how would you rate your physical health in general now?	1= Much better<br>2= Slightly better<br>3= About the same<br>4= Slightly worse<br>5= Much worse
**H9**	RAND-12	Compared to one year ago, how would you rate your emotional problems (such as feeling anxious, depressed or irritable) now?	1= Much better<br>2= Slightly better<br>3= About the same<br>4= Slightly worse<br>5= Much worse
**N/A**	Transition	Over the last two weeks, have you felt bothered by the following items?	N/A (transition)
**H10**	GAD-2	Feeling nervous, anxious, or on edge.	1= Not at all<br>2= Several days<br>3= More than half the days<br>4= Nearly every day
**H11**	GAD-2	Not being able to stop or control worrying.	1= Not at all<br>2= Several days<br>3= More than half the days<br>4= Nearly every day
**H12**	PHQ-2	Little pleasure or interest in doing things.	1= Not at all<br>2=Several days

Jesse Brown VAMC IRB
Approval Date: December 23, 2020

			3= More than half the days<br>4= Nearly everyday
**H13**	PHQ-2	Feeling down, depressed, or hopeless.	1= Not at all<br>2=Several days<br>3= More than half the days<br>4= Nearly everyday
**Awareness and Knowledge about COVID-19**			
**N/A**	Transition	Now, I am going to ask questions specific to the COVID-19 pandemic.	N/A (transition)
**AK1**	HBM: Perceived knowledge	How much would you say you know about the COVID-19 pandemic?	1= Nothing at all<br>2= A little<br>3= A moderate amount<br>4 = A lot
**AK2**	Ecological; intrapersonal; knowledge	Which of the following do you think can be symptoms of COVID-19? Select all that apply.	1= Fever<br>2=Cough<br>3=Shortness of breath<br>4= Runny of stuffy nose<br>5=Muscle or body aches<br>6= Headaches<br>7= Fatigue<br>8= Rash on face<br>9= Vomiting or diarrhea<br>10= Chest Pain<br>11= Ear Pain<br>12= Sore throat<br>13= Loss of taste or smell<br>14 =Unsure
**AK3**	Ecological; intrapersonal; knowledge	Persons over the age of 65 are at greater risk of severe illness if they get COVID-19.	1= Strongly disagree<br>2= Disagree<br>3= Not sure<br>4= Agree<br>5= Strongly agree
**AK4**	Ecological; intrapersonal; knowledge	COVID-19 can be spread from person to person.	1= Strongly disagree<br>2= Disagree<br>3= Not sure<br>4= Agree<br>5= Strongly agree
**AK5**	Ecological; intrapersonal; knowledge	People with COVID-19 always show symptoms.	1= Strongly disagree<br>2= Disagree<br>3= Not sure<br>4= Agree<br>5= Strongly agree
**AK6**	Ecological; intrapersonal; knowledge	Most people who get COVID-19 only show mild symptoms?	1= Strongly disagree<br>2= Disagree<br>3= Not sure<br>4= Agree<br>5= Strongly agree
**AK7**	Ecological; intrapersonal;	After a person has recovered form COVID-	1= Strongly disagree<br>2=Disagree

Jesse Brown VAMC IRB
Approval Date: December 23, 2020

	knowledge	19, he/she <u>cannot</u> get it again.	3= Agree<br>4= Strongly agree
**Attitude & Beliefs**			
**AB1**	HBM: Perceived risk	What do you think is the chance you will get COVID-19 in the future?	1= No chance<br>2= Low chance<br>3= Moderate Chance<br>4= High chance
**AB2**	HBM: Perceived severity	How sick do you think you would be if you got COVID-19?	1= Not sick at all<br>2= A little sick<br>3= Moderately sick<br>4= Very sick
**Vaccination**			
**N/A**	Transition	The following questions are going ask about your views on vaccination.	N/A (transition)
**V1**	Ecological; intrapersonal; knowledge	I believe vaccines are safe for the general population.	1= Strongly disagree<br>2= Disagree<br>3= Agree<br>4= Strongly Agree
**V2**	Ecological; intrapersonal; knowledge	I believe there is enough information about vaccines and their safety.	1= Strongly disagree<br>2= Disagree<br>3= Agree<br>4= Strongly agree
**V3**	HBM: Perceived benefit	Vaccination decrease the chance of infections.	1= Strongly disagree<br>2= Disagree<br>3= Agree<br>4= Strongly agree
**V4**	HBM: Perceived benefit	Vaccination decrease the severity of disease if infected.	1= Strongly disagree<br>2= Disagree<br>3= Agree<br>4= Strongly Agree
**V5**	HMB: Perceived benefit	Vaccinations make me feel less worried about becoming infected with diseases.	1= Strongly disagree<br>2= Disagree<br>3= Agree<br>4= Strongly agree
**V6**	Ecological: Intrapersonal; Knowledge	New vaccines carry more risks than older vaccines.	1= Strongly disagree<br>2= Disagree<br>3= Not sure<br>4= Agree<br>5= Strongly agree
**V7**	HBM: Perceived barriers	Which of the following represent your specific concerns about vaccine safety?	1= Side effects<br>2= Efficacy (does it work)<br>3= Newness—not wanting to be the first<br>4= Rigor/thoroughness of testing<br>5= Vaccine contents<br>6= I do not have any concerns

Jesse Brown VAMC IRB
Approval Date: December 23, 2020

**V8**	Acceptance (general)	I receive the annual flu shot.	1= Never<br>2= Some years<br>3= Every year	
**V9**	Acceptance	When a vaccine for COVID-19 becomes readily available at the VA, I will get it.	1= Strongly disagree<br>2= Disagree<br>3= Agree<br>4= Strongly agree	
**N/A**	HBM: Perceived barriers	How much, if at all, would each of the following influence whether you, personally, would get a vaccine to prevent COVID-19?	N/A; transition	
**V10**	HBM: Perceived barriers	Many people report soreness at vaccination site.	1= Would make me A LOT less likely to get a vaccine<br>2= Would make me A LITTLE less likely to get a vaccine<br>3= Would not make a difference	
**V11**	HBM: Perceived barriers	If you need to get the vaccine for COVID-19 every year.	1= Would make me A LOT less likely to get a vaccine<br>2= Would make me A LITTLE less likely to get a vaccine<br>3= Would not make a difference	
**V12**	HBM: Cue to action	If my doctor or other health care provider recommends the vaccine for COVID-19 to me, I will get it.	1= Strongly disagree<br>2= Disagree<br>3= Agree<br>4= Strongly agree	
**V13**	HBM: Cue to action	If a religious leader recommends the vaccine for COVID-19, I will get it.	1= Strongly disagree<br>2= Disagree<br>3= Agree<br>4= Strongly agree	
**V14**	HBM: Cue to action	If a family member, recommends the vaccine for COVID-19, I will get it.	1= Strongly disagree<br>2= Disagree<br>4= Agree<br>5= Strongly agree	
**V15**	HBM: Cue to Action	Which of the other following people would influence you to get the vaccine for COVID-19 if you see them get it on the news?	1= Community leader<br>2= Governor<br>3= Mayor<br>4= Friends<br>5= Scientists (i.e. Dr. Fauci)<br><br>6= Current or Prior US	Select all that apply.

Jesse Brown VAMC IRB
Approval Date: December 23, 2020

			Presidents (Biden, Trump, Obama, Clinton, etc.)<br>7=None	
**V16**	Ecological; Interpersonal; Acceptance	I will recommend to family members and friends to get the COVID-19 vaccine once it becomes available.	1= Strongly disagree<br>2= Disagree<br>3= Agree<br>4= Strongly agree	
**Trust**				
N/A	Transition	The following questions will ask about how much your trust your health care provider and the VA in general.	N/A	
**T1**	Ecological; community; Trust (system)	How much trust do you have in government with respect to fighting the COVID-19 pandemic?	1= None at all<br>2= A little<br>3= Moderate<br>4= Complete	
**T2**	Ecological; community; Trust (system)	How much trust do you generally have in the information provided by the government about the COVID-19 pandemic?	1= None at all<br>2= A little<br>3= Moderate<br>4= Complete	
**T3**	Ecological; community; Trust (system)	Which of the following people/organizations do you trust?	1= Your own doctor<br>2= Health care professionals (i.e. doctors, nurses, paramedics, and pharmacists)<br>3= Local/County health<br>4= State health department<br>5= Centers for Disease Control (CDC)<br>6= Food and Drug Administration<br>7= The White House<br>8=Congress<br>9= Department of Health and Human Services<br>10= National Institute of Health<br>11= Scientists<br>12= None	(Select all that apply)
**N/A**	Transition	The next set of questions are about your health care provider. Please respond to these questions as it pertains your regular doctor or if you do not have one,		

Jesse Brown VAMC IRB
Approval Date: December 23, 2020

		refer to your last health visit.		
**T4**	Ecological; community; Physician Trust Survey	I trust my doctor's decisions about which treatments are best for me.	1= None of the time<br>2= A little of the time<br>3 = Most of the time<br>4= Always	
**T5**	Ecological; community; Physician Trust Survey	My doctor is extremely thorough and careful.	1= None of the time<br>2= A little of the time<br>3 = Most of the time<br>4= Always	
**T6**	Ecological; community; Physician Trust Survey	Sometimes my doctor cares more about what is convenient for (him/her).	1= None of the time<br>2= A little of the time<br>3 = Most of the time<br>4= Always	
**T7**	Ecological; community; Physician Trust Survey	My doctor is completely honest with me.	1= None of the time<br>2= A little of the time<br>3 = Most of the time<br>4= Always	
**T8**	Ecological; community; Physician Trust Survey	I have complete trust in my doctor.	1= None of the time<br>2= A little of the time<br>3 = Most of the time<br>4= Always	
**N/A**	Transition	The following questions pertain to your trust in the VA health system.		
**T9**	VA Trust Survey	I trust the VA to put my medical needs above all other things.	1= None of the time<br>2= A little of the time<br>3 = Most of the time<br>4= Always	
**T10**	VA Trust Survey	The medical skills of the VA doctors and nurses are NOT as good as they should be.	1= None of the time<br>2= A little of the time<br>3 = Most of the time<br>4= Always	
**T11**	VA Trust Survey	I always trust the VA to give me the information I need about my treatment.	1= None of the time<br>2= A little of the time<br>3 = Most of the time<br>4= Always	
**T12**	VA Trust Survey	The VA will not give me the best possible care.	1= None of the time<br>2= A little of the time<br>3 = Most of the time<br>4= Always	
**Source of Information/Communication**				
**I1**	Ecological; community; Information	What is your primary source(s) of information for the COVID-19 pandemic?	1= Work/employer<br>3= Television/radio<br>4= Internet<br>5= Friends or family<br>6= Other, please specify:	
**I3**	Communication	How would you like to learn about a new COVID-19 vaccine?	1= In a fact sheet<br>2= Through a question answer format	Select all that apply

Jesse Brown VAMC IRB
Approval Date: December 23, 2020

	Please select all that apply.	3= By having people who have taken it share their experience<br>4= Doctor, nurse, or other health care provider communication<br>5= Some other way, please tell us: __	
**Demographics**			
**Construct**	**Item**	**Response Scale**	**Notes**
**Demographics**	What is your age?	_____ years old	
**Demographics**	What is the highest level of education that you have completed?	1=1st to 8th grade<br>2=Some high school, no diploma<br>3=High school graduate, diploma or the equivalent (for example: GED)<br>4=Some college credit, no degree<br>5=Trade/technical/vocational training<br>6=Associate degree<br>7=Bachelor's degree<br>8=Master's degree<br>9=PhD or Doctorate degree	
**Additional demographics**			
**Ecological; community**	How would you classify your residence?	1= Single family home<br>2= 2-6 units in a building<br>3= Large apartment complex<br>4 = Other	
**Ecological; community**	What is your current living situation?	1= Live alone<br>2= Live with one other<br>3= Live with two or more	
**Ecological; community**	What is your primary source of transportation (that you use the most)?	1= Public transportation<br>2= Taxi, MediCar, Lyft, Uber, etc.<br>3= Personal Car<br>4= Walk<br>5= Bike<br>6= Other	
**Follow-up**			
**F1**	Do you give permission for us to call you to ask if you will take another survey or participate in an interview?	1=Yes<br>2=No	

Jesse Brown VAMC IRB
Approval Date: December 23, 2020
