# Supplementary material for: An assessment of Veterans attitudes and willingness to receiving the COVID-19 vaccine: a mixed methods study
Source: BMC Infect Dis. 2022 Mar 29;22:308. doi: 10.1186/s12879-022-07269-7 (PMC8961481; doi:10.1186/s12879-022-07269-7)
Supplement: Supplementary file 2 — Additional file 2: In-depth interview guiding questions. [file 12879_2022_7269_MOESM2_ESM.docx]

COVID-19 Registry: Sub Study 8 Telephone Script & Interview Guide

Hello. May I speak to __________ (patient’s name)?

Once patient is reached:

Good morning/afternoon, evening Mr./Ms._______, this is _________ from the Jesse Brown VA. Can I verify your date of birth please? Once patient’s DOB verified: I’m calling you because we you recently completed a telephone survey on the COVID-19 vaccine and gave us permission to call you to ask if you would like to take part in an additional interview.

Is this a good time to talk about the study?

If **No**: What day and time would be more convenient for us to call you back? Thank you and I’ll talk to you again on (date/time).

If **Yes**: Before I give you an overview of what the interview is about and why we contacted you, let me emphasize that this call is an invitation to participate in the interview and your involvement is completely voluntary.

[Give overview of study, including a brief summary of what would be asked of him/her.]

Let me answer any questions you may have. [Pause for questions and address them].

This is a one-time interview that will last between 30-60 minutes and will be audio-recorded to ensure accuracy of information collected and any person identifiers will be removed from the transcript. You may decline to answer any question you do not want to answer. We are going to ask you some questions about getting a vaccine. Sometimes a vaccine is also called a “shot” – like the “flu shot” or a “tetanus shot”. Vaccines can be once in your life – like a measles, mumps, rubella shot (MMR) that children get – or can be once a year like the flu shot.

Do you agree to participate in this study knowing that the interview voluntary and that you can withdraw at any point with no consequences to you? If yes: is it ok if we begin now?

1. Tell me how has COVID-19 affected your life since it was declared a pandemic in March of 2020?
   1. What kind of preventative measures have you taken to protect yourself or others?
   2. How has the COVID-19 pandemic affected your employment?
   3. How has the pandemic affected your physical health? (Your mental health?)
2. Tell me about your experience with getting vaccines.
3. What are some reasons why you may agree to get or not get a vaccine? (e.g. annual flu vaccine, tetanus, or Shingles?)
   1. Why would you agree or not?
   2. What side effects have your heard about vaccines? Which side effects concern you the most?
   3. What are the benefits of vaccination/what are the risks?
4. Have you heard rumors about vaccines?
   1. What rumors have you heard about vaccines?
   2. What rumors have your heard about the COVID-19 vaccine?
   3. Where do you get most of your information about the COVID-19 vaccine?
5. What do you know about the new COVID-19 vaccines?
   1. What is the first thing you want to know about the new COVID-19 vaccines? (e.g. safety, having to wait/not in “at-risk” groups, questions about it, or lack of trust in government)
   2. Tell me what you’ll do when a vaccine for COVID-19 is available to you at the VA?
   3. What are some reasons why you would/or would not get the COVID-19 vaccine?
   4. What do you think about getting the COVID-19 vaccine in order to protect others at risk?
   5. Compare how you feel about the new COVID-19 vaccines to other vaccines (e.g. flu, tetanus, Shingles).
6. [If Veteran indicates he/she may get vaccine at a later date] What information would change your mind about getting the COVID-19 vaccine?
7. What else do you want to share about getting/not getting a COVID-19 vaccine?
