# Supplementary material for: An assessment of Veterans attitudes and willingness to receiving the COVID-19 vaccine: a mixed methods study
Source: BMC Infect Dis. 2022 Mar 29;22:308. doi: 10.1186/s12879-022-07269-7 (PMC8961481; doi:10.1186/s12879-022-07269-7)
Supplement: Supplementary file 3 — Additional file 3: Reporting guidelines for Standard for Reporting Qualitative Research (SRQR). [file 12879_2022_7269_MOESM3_ESM.docx]

Supplemental Table 1. SRQR Reporting checklist for Qualitative Studies

|  |  | Reporting Item | Section (Page No.) |
| --- | --- | --- | --- |
| Title |  |  |  |
|  | #1 | Concise description of the nature and topic of the study identifying the study as qualitative or indicating the approach (e.g., ethnography, grounded theory) or data collection methods (e.g., interview, focus group) is recommended | Title (p. 1) |
| Abstract | | | |
|  | #2 | Summary of key elements of the study using the abstract format of the intended publication; typically includes background, purpose, methods, results and conclusions. | Abstract (p. 2-3) |
| Introduction | | | |
| Problem formulation | #3 | Description and significance of the problem / phenomenon studied: review of relevant theory and empirical framework; problem statement | Introduction (p. 4) |
| Purpose or research question | #4 | Purpose of the study and specific objectives or questions | Introduction (p. 4) |
| Methods | | | |
| Qualitative approach and research paradigm | #5 | Qualitative approach (e.g., ethnography, grounded theory, case study, phenomenology, narrative research) and guiding theory if appropriate; identifying the research paradigm (e.g., postpositivist, constructivist / interpretivist) is also recommended; rationale. The rationale should briefly discuss the justification for choosing that theory approach, methods, or technique rather than other options available; the assumptions and limitation implicit in those choices and how those choices influence study conclusions and transferability. As appropriate the rationale for several items might be discussed together. | Methods (p. 5-8) |
| Researcher characteristics and reflexivity | #6 | Researchers’ characteristics that may influence the research including personal attributes, qualifications / experience, relationship with participants, assumptions and / or presuppositions; potential or actual interactions between researchers’’ characteristics and the research questions, approach, methods, results and / or transferability. | Methods (p. 8) |
| Context | #7 | Setting / site and salient contextual factors; rationale | Methods (p. 5-6) |
| Sampling strategy | #8 | How and why research participants, documents, or events were selected: criteria for deciding when no further sampling was necessary (e.g., sampling saturation): rationale | Methods p. 5-6) |
| Ethical issues pertaining to human subjects | #9 | Documentation of approval by an appropriate ethics review board and participant consent, or explanations for lack thereof; other confidentiality and data security issues | Methods (p. 5-6) |
| Data collection methods | #10 | Description of instruments (e.g., interview guides, questionnaires) and devices (e.g., audio recorders) used for data collection; if / how instruments(s) changed over the course of the study | Methods (p. 5-7) |
| Units of study | #11 | Number and relevant characteristics of participants documents, or events included in the study; level of participation (could be reported in results) | Methods (p. 5, 9-10)  Results (p. 8&11) |
| Data processing | #12 | Methods for processing data prior to and during analysis, including transcription, data entry, data management and security, verification of data integrity, data coding and anonymization / deidentification of excerpts | Methods (p. 8) |
| Data analysis | #13 | Process by which interferences, themes, etc. were identified and developed, including the researchers involved in data analysis; usually references a specific paradigm or approach; rationale | Methods (p. 7-8 & p.18-19) |
| Techniques to enhance trustworthiness | #15 | Technique to enhance trustworthiness and credibility od data analysis (e.g., member checking, audit trial, triangulation); rationale | Methods (p. 8) |
| Results/findings | | | |
| Synthesis and interpretation | #16 | Main findings (e.g., interpretations, inferences, and themes); might include development of a theory or model, or integration with prior research or theory | Results (pp. 9-12) |
| Links to empirical data | #17 | Evidence (e.g., quotes, field notes, texts excerpts, photographs) to substantiate analytic findings | Results (pp.11-12) |
| Discussion | | | |
| Integration with prior work, complications, transferability and contribution(s) to the field | #18 | Short summary of main findings; explanation of how findings and conclusion connect to, support, elaborate on, or challenge conclusion of earlier scholarship; discussion of scope of application / generalizability; identification of unique contributions(s) to scholarship in a discipline or field | Discussion (pp.13-16) |
| Limitations | #19 | Trustworthiness and limitations of findings | Discussion (p. 16) |
| Other | | | |
| Conflicts of interest | #20 | Potential sources of influence of perceived influence on study conduct and conclusions; how these were managed | Back materials (p. 18) |
| Funding | #21 | Sources of funding and other support; role of funders in data collection, interpretation and reporting | Back materials (p. 18) |
